# Optimal weights and priors in simultaneous fitting of multiple small-angle scattering datasets

**DOI:** 10.1107/S1600576725002390

**Published:** 2025-05-02

**Authors:** Andreas Haahr Larsen

**Affiliations:** a University of Copenhagen Niels Bohr Institute Universitetsparken 5 2100 Copenhagen Denmark; Argonne National Laboratory, USA

**Keywords:** co-refinement, small-angle scattering, SAXS, SANS, simultaneous fitting, Bayesian refinement, weighting schemes, priors

## Abstract

Small-angle X-ray and neutron scattering (SAXS and SANS) are powerful techniques in materials science and soft matter. This study addressed how multiple SAXS or SANS datasets are best weighted when performing simultaneous fitting. Three weighting schemes were tested: (1) equal weighting of all datapoints, (2) equal weighting of each dataset through normalization with the number of datapoints and (3) weighting proportional to the information content. The weighting schemes were assessed by model refinement against synthetic data under numerous conditions. The first weighting scheme led to the most accurate parameter estimation, especially when one dataset substantially outnumbered the other(s). Furthermore, it was demonstrated that inclusion of Gaussian priors significantly improves the accuracy of the refined parameters, as compared with common practice, where each parameter is constrained uniformly within an allowed interval.

## Abstract 2

Here, we determine the optimal weights and priors to use when simultaneously fitting small-angle X-ray and neutron scattering data.

## 1. Introduction

Small-angle X-ray and neutron scattering (SAXS and SANS) provide structural information about nanoscale structures, ranging from a few to hundreds of nanometres. They have applications across diverse fields, including investigations of amorphous materials like gels, polymers and glasses, as well as biological macromolecules such as proteins, DNA, lipids and their complexes. Hard materials, including nanoparticles, also fall within the scope of investigation. By combining SAXS or SANS measurements that have different scattering-length contrasts, structural domains can be highlighted, resulting in more accurate refinement of structural parameters.

Contrast variation can be achieved in SAXS by changing the ionic strength of the solvent (Gabel *et al.*, 2019 ▸), and in SANS the contrast can be varied using hydrogen–deuterium exchange in sample or solvent (Heller, 2010 ▸). SAXS and SANS have elegantly been combined, *e.g.* in studies of toroidal polymer assemblies (Hollamby *et al.*, 2016 ▸), protein/DNA complexes (Sonntag *et al.*, 2017 ▸), multishell nanoparticles (Lin *et al.*, 2020 ▸), growing gold nanorods (Zech *et al.*, 2022 ▸), block copolymer micelles (Manet *et al.*, 2011 ▸), multilamellar lipid vesicles (Heftberger *et al.*, 2014 ▸) and lipid nanodiscs (Kynde *et al.*, 2014 ▸), to mention a few examples. However, choosing proper weights to each dataset is not trivial: should one simply weight with the number of points and their respective errors, or should the number of points be normalized out in the minimization? Should the noise level and information content be taken into account in the minimization algorithm? In this paper, three weighting schemes were compared: (1) a naive weighting scheme, where each datapoint is weighted according to its statistical uncertainty, meaning that datasets with more points and smaller errors have more weight; (2) a reduced weighting scheme, where each dataset is given equal weight, corresponding to minimizing the reduced χ^2^; and (3) an information-based weighting scheme, where each dataset is weighted proportional to its information content. Model parameters were co-refined against synthetic data, and the refined values were compared with the known ground truth to evaluate and compare the different weighting schemes.

Another central aspect in modeling is the inclusion of molecular constraints (Zemb & Diat, 2010 ▸) or prior knowledge. The present study tests the use of Bayesian refinement with Gaussian priors for enhanced accuracy in co-refinement against multiple SAXS or SANS datasets. This is inspired by successful applications of Bayesian refinement in X-ray crystallography (Headd *et al.*, 2012 ▸), electron microscopy (Scheres, 2012*a* ▸,*b* ▸) and reflectometry (Nelson & Prescott, 2019 ▸; McCluskey *et al.*, 2020 ▸, 2023 ▸), and for the combining of SAXS with molecular dynamics simulations (Hummer & Köfinger, 2015 ▸).

## 2. Methods

This paper relies on fitting simulated or synthetic data. Thus, the ground truth is known, allowing for quantitative evaluation of different weighting schemes and prior inclusion. For the generation and analysis of synthetic data, two form factors were applied.

### 2.1. Core–multishell form factor

**Figure 1:**
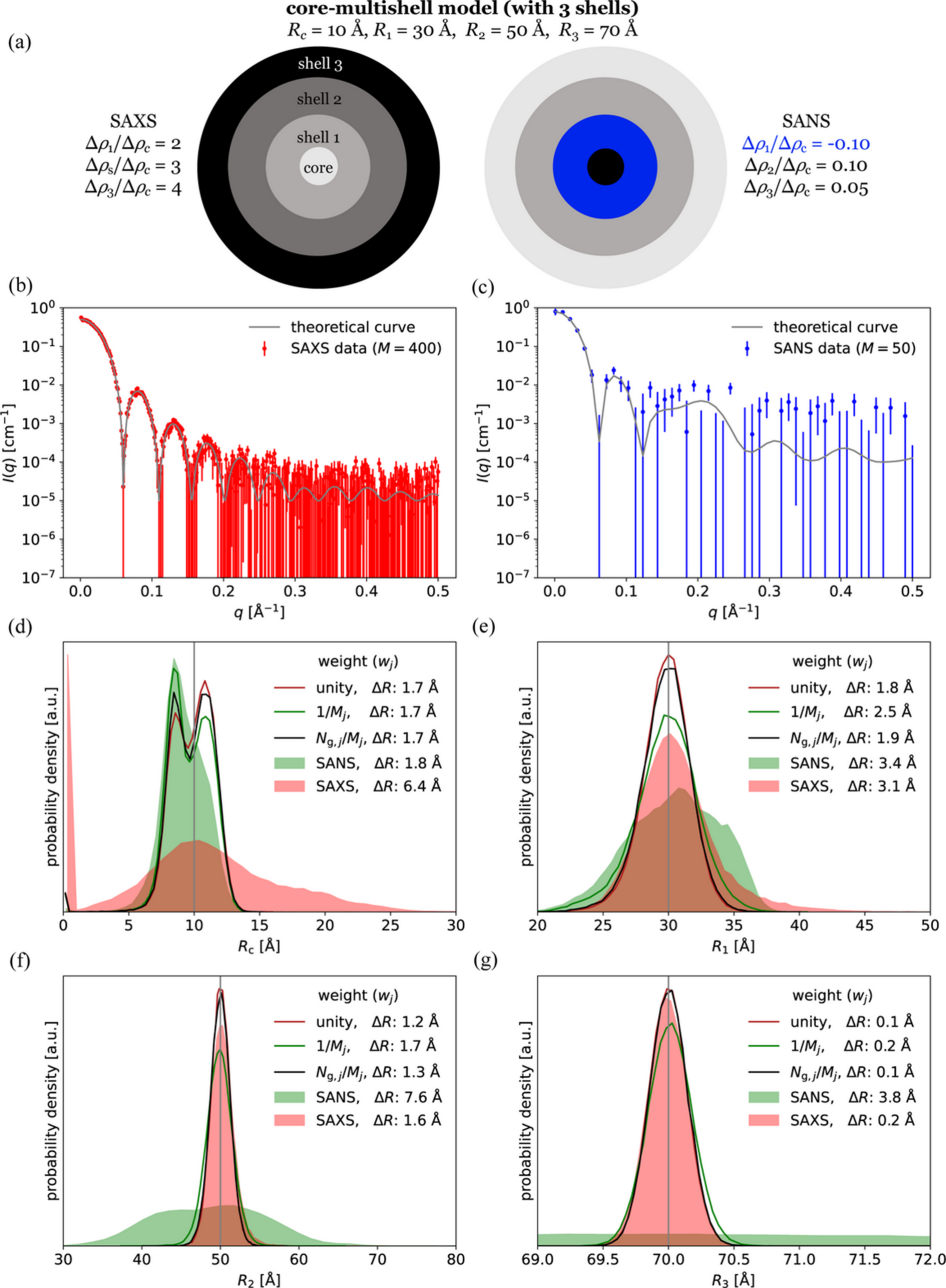
Refinement of a core–multishell model using different weighting schemes. (*a*) Core–multishell particle with relative scattering contrasts and radii annotated. (*b*) Simulated SAXS-like data with 400 points. (*c*) Simulated SANS-like data with 50 points. (*d*)–(*g*) Refined values of *R*_c_, *R*_1_, *R*_2_ and *R*_3_ from 50000 fits (new data simulated each time). The parameters were refined against SANS alone (green area), SAXS alone (red area), or SAXS and SANS with the naive weighting scheme (red line), the reduced weighting scheme (green line) or the information-based weighting scheme (red line). The gray vertical line is the true value.

The core–shell model is built up using the form-factor amplitude for a sphere with radius *R*: 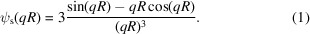
The amplitude of the scattering vector is 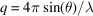, where 2θ is the scattering angle and λ is the wavelength of the incoming wave. The volume of the sphere is *V*_s_(*R*) = 4π*R*^3^/3. The core radius of the model is denoted *R*_c_ and the outer radius of the *j*th shell is denoted *R*_*j*_. The difference in scattering-length density between the *j*th shell and the solvent, *i.e.* the scattering contrast, is Δρ_*j*_. The form factor for a core–multishell particle with *n*_s_ shells can be written as 
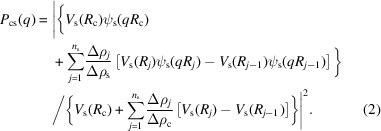
For this article, we used three shells (*n*_s_ = 3), as illustrated in Fig. 1. The intensity is modeled with a scaling factor, *a*, and a constant background, *b*, as *I*_cs_(*q*) = *aP*_cs_(*q*) + *b*. Only the relative values of the individual contrasts affect *P*(*q*), so the model has nine parameters (*K* = 9): four radii (*R*_c_, *R*_1_, *R*_2_, *R*_3_) and three relative scattering contrasts (Δρ_*j*_/Δρ_c_), as well as the scaling factor and the constant background. When fitting two datasets with the model, five additional parameters were introduced, namely three relative scattering contrasts, scaling and background for the second dataset (*K* = 14).

### 2.2. Stacked-cylinder form factor

**Figure 2:**
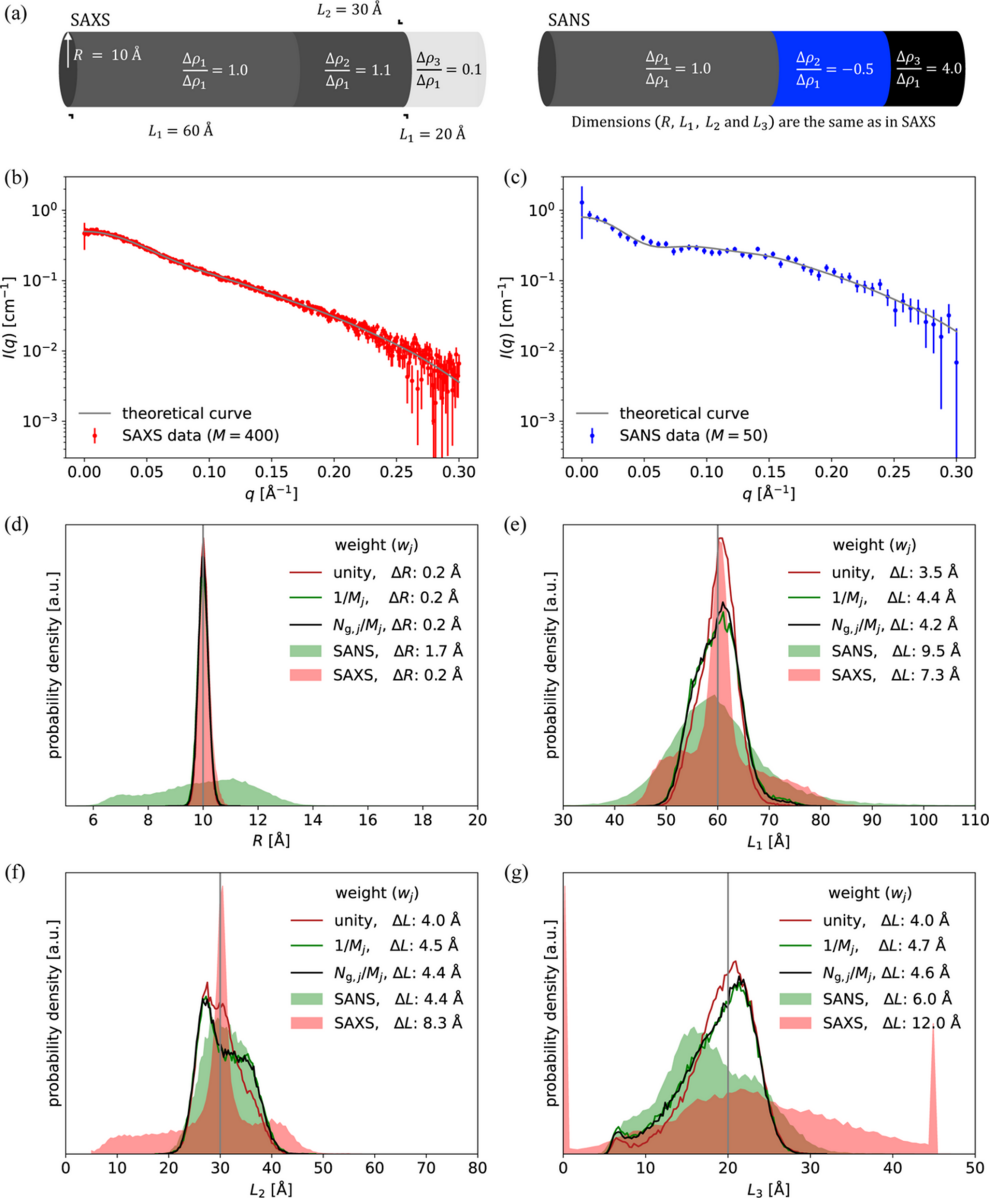
Refinement of a stacked-cylinder model against simulated data, using different weighting schemes. (*a*) Stacked cylinders with dimensions and relative scattering contrasts annotated. (*b*) Simulated SAXS-like data with 400 points. (*c*) Simulated SANS-like data with 50 points. (*d*)–(*g*) Histograms of refined values of *R*, *L*_1_, *L*_2_ and *L*_3_ (gray line is the true value), after simultaneous fits to 50000 pairs of simulated SAXS and SANS data.

For testing the method against a less symmetric model with a different contrast situation, a stacked-cylinder form factor was used. The model is based on the form-factor amplitude for cylinders with radius *R* and length *L* (Pedersen, 1997 ▸):
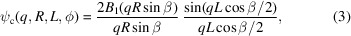
where *B*_1_ is the first-order Bessel function of the first kind. The scattering depends on the cylinder orientation, as described by the angle β, so the form-factor amplitude should be integrated over β to yield the cylinder form factor for a sample of non-oriented cylinders. The volume of the cylinder is *V*_c_(*R*, *L*) = π*R*^2^*L*. The form factor for *n*_c_ stacked cylinders with radii *R*_*j*_, lengths *L*_*j*_ and scattering contrasts Δρ_*j*_ is 
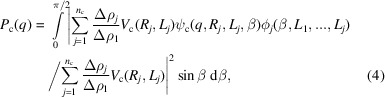
where ϕ_*j*_ is the phase factor of the *j*th cylinder, which depends on the center-to-center distance to the first cylinder: 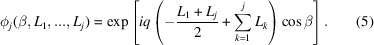
In the special case *j* = 1, ϕ_*j*_ is unity. For this article, we used three stacked cylinders (*n*_c_ = 3), each with the same radius but with varying lengths, as illustrated in Fig. 2. The intensity was modeled with a scale and a background, *I*_c_(*q*) = *aP*_c_(*q*) + *b*. This model had seven parameters (*K* = 7) when refined against a single dataset, and 11 parameters (*K* = 11) when two datasets were simultaneously fitted.

### 2.3. Model implementation and validation

The form factors were implemented in *BayesFit* (https://github.com/andreashlarsen/BayesFit) and validated against simulated data generated in *Shape2SAS* (Larsen *et al.*, 2023 ▸).

### 2.4. Simulated SAXS and SANS data

First, the *q* range was defined, with *q*_min_ = 0.001 Å^−1^ and *q*_max_ = 0.5 Å^−1^ for the spherical core–multishell particles, and *q*_min_ = 0.0001 Å^−1^ and *q*_max_ = 0.3 Å^−1^ for the stacked cylinders. The simulated SANS-like data contained 50 or 300 uniformly distributed points, and the simulated SAXS-like data contained either 300, 400, 900 or 2000 points. Theoretical curves were then calculated and evaluated at these *q* values, using *I*_model_(*q*) = *aP*(*q*) + *b*. The SAXS data were scaled by *a*_SAXS_ = 0.5 cm^−1^ and the SANS data by *a*_SANS_ = 0.8 cm^−1^, and a constant background of *b* = 10^−5^ cm^−1^ was added to the SAXS data and *b* = 10^−4^ cm^−1^ was added to the SANS data. The higher background in the simulated SANS data reflects incoherent scattering. To ensure realistic errors, similar to what would be obtained from an experiment, the errors were modeled using an empirical model (Sedlak *et al.*, 2017 ▸): 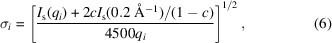
where *I*_s_(*q*_*i*_) = *sI*_model_(*q*_*i*_)/*I*(0) is the normalized and scaled model intensity evaluated at *q*_*i*_, and σ_*i*_ are the standard deviations, which in an experiment are estimated through counting statistics and error propagation. The absolute intensity is scaled to realistic values by the factor *s*, and the empirical constant *c* relates the buffer intensity to the sample intensity (Sedlak *et al.*, 2017 ▸). For simulated SAXS-like data, *s* = 100 and *c* = 0.85 were used, whereas for simulated SANS-like data, *s* = 10 and *c* = 0.95 were used. These values were chosen to reflect typical SAXS or SANS data, and the simulated SAXS data had a higher signal-to-noise ratio to reflect higher flux compared with the simulated SANS data. The simulated intensities (*I*_*i*_) were then pulled stochastically from normal distributions with mean μ_*i*_ = *I*_model_(*q*_*i*_) and standard deviation σ_*i*_.

To simulate data with increased noise, the variance (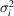) was multiplied with a noise factor before simulation of the intensities, *i.e.*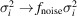. The noise was increased logarithmically, by varying 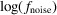 from −4 to 10. In order to simulate data with over- or under-estimated errors, σ_*i*_ was multiplied by a factor after simulation of the data, such that the new σ_*i*_ no longer reflected the fluctuations of the simulated intensities (Smales & Pauw, 2021 ▸; Larsen & Pedersen, 2021 ▸). For each condition, *i.e.* the different weight schemes and priors described in the *Results*[Sec sec3], 50000 SAXS and 50000 SANS datasets were simulated and fitted with the model.

Due to wavelength spread, divergence and pixel size, there are instrumental smearing effects or resolution effects (Pedersen *et al.*, 1990 ▸). These are usually negligible in synchrotron SAXS data, but not in SANS and laboratory-source SAXS data. Depending on the instrumental settings, the resolution effects can, in many cases, be expressed as a normal distributed error, σ_*q*_, for each *q* value and included in the model by smearing the theoretical intensity: 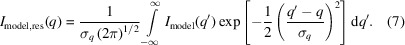
At many SANS instruments, the values of σ_*q*_ are provided as a fourth column in the datafile. To investigate the effect of smearing, the fourth column of a SANS dataset from D22 was used [Small Angle Scattering Biological Data Bank (SASBDB; Kikhney *et al.*, 2020 ▸) entry SASDL53; Lycksell *et al.*, 2021 ▸]. These data were measured with SEC–SANS (size-exclusion chromatography–SANS) at two sample-to-detector distances of 2.8 and 11.2 m, which were merged. The wavelength (λ) was 6 Å, with a relative resolution (Δλ/λ) of 10%. The experimental σ_*q*_ values were imported and linearly interpolated to the simulated *q* values. The resolution effects were taken into account when fitting these data, using the same σ_*q*_ values that were used to simulate the data. In order to investigate more influential resolution effects, data were also simulated with σ_*q*_ multiplied by a factor of 2 or 3 and fitted using these values.

### 2.5. *BayesFit* – fitting multiple datasets with priors

*BayesFit* is a pro­gram that can fit SAXS and SANS data simultaneously with an analytical model and use Gaussian priors. Priors are probability distributions for the values of the model parameters, *e.g.* the concentration, the scattering-length densities or the geometrical parameters. The priors are based on knowledge obtained before modeling of the SAXS or SANS data and therefore provide complementary structural information. *BayesFit* was originally implemented in Fortran (Larsen *et al.*, 2018 ▸). For this paper, a new implementation was written in Python, to facilitate fitting of multiple datasets. *BayesFit* reads an input file, which contains information about the data, the name of the model, the prior values for each model parameter (μ_prior,*k*_ and σ_prior,*k*_) and the weights (*w*_*j*_) used to balance different datasets. The weight given to the prior is adjusted by a hyperparameter, α (Hansen, 2000 ▸; Larsen *et al.*, 2018 ▸). *BayesFit* minimizes
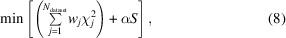
where χ^2^ and *S* are given as 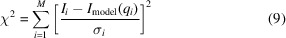
and 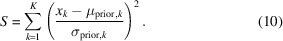
The terms μ_prior,*k*_ and σ_prior,*k*_ are the mean and standard deviation of the prior distribution for the *k*th model parameter, *x*_*k*_ is the refined value, and *M* is the number of datapoints. The prior weights in equation (8) were adjusted by a regularization parameter, α, which is determined by maximizing the probability of the refined solution (Hansen, 2000 ▸; Larsen *et al.*, 2018 ▸). For the refinements in this paper, *BayesFit* scanned 11 logarithmically spaced values of α and the range was manually adjusted to ensure that it contained the α values giving rise to the highest probabilities. This was done by plotting the probabilities for a series of α values around 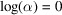, *e.g.* from 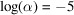 to 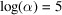. The range should contain the maximum for the probability and converge to zero at the minimum and maximum. If not, the range was adjusted. *BayesFit* utilizes *Scipy*’s curve_fit function (Virtanen *et al.*, 2020 ▸). In order to use the curve_fit function, an array was defined with all *q* values from both SAXS and SANS data and dummy *q* values for each of the prior values. A corresponding array was defined with all simulated intensities (*I*_*i*_) from the SAXS and SANS datasets and the prior means (μ_prior,*k*_). Finally, an array was con­structed with the errors of the simulated data (σ_*i*_) as well as the prior standard deviations (σ_prior,*k*_). The experimental errors were scaled with 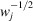 before fitting, to obtain the weighting in equation (8). The prior means (μ_prior,*k*_) were used as initial guesses in the subsequent nonlinear minimization. The upper and lower limits were set to  ± 5σ_prior,*k*_, and parameters were constrained to positive values when relevant. To apply uniform priors, α was fixed at 10^−10^, effectively quenching the effect of the prior, except for the upper and lower limits, which were adjusted by changing σ_prior,*k*_. The means, μ_prior,*k*_, were also used as initial guesses when fitting with uniform priors. Parameter values for all priors are listed in Tables 2 and 3. Normalized Hessian matrices and their eigenvalues were used to calculate the information content (Vestergaard & Hansen, 2006 ▸). The Hessian matrices were constructed numerically from χ^2^ using the forward Euler method, and eigenvalues were found using *NumPy* (Harris *et al.*, 2020 ▸). The total probability of the solution, taking into account the likelihood and priors, was derived from Bayes theorem (Hansen, 2000 ▸; Larsen *et al.*, 2018 ▸). Each refined model parameter was then calculated as a probability-weighted average: 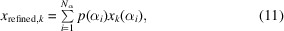
where *p*(α_*i*_) is the probability density of the solution at α_*i*_ and *x*_*k*_(α_*i*_) is the refined value of the *k*th parameter at α_*i*_. *N*_α_ is the number of α values that were scanned. The program is meant as a proof of concept, and the goal is that inclusion of Gaussian priors and optimal weighting should be implemented in other software packages for SAXS and SANS analysis that are superior in the number of verified models, user interface, performance and additional features. Such programs include *WillItFit* (Pedersen *et al.*, 2013 ▸) and *SasView* (https://www.sasview.org). From *SasView* version 6, it was made possible to adjust weights in simultaneous fitting (https://www.sasview.org/downloads/modifying_weights_in_sasview_v6.pdf), which calls for thorough investigations of which weighting scheme is most optimal.

### 2.6. Calculating information content

The number of good parameters (*N*_g,BIFT_) was used as a measure for the information content in data. *N*_g,BIFT_ is an estimate of the number of independent parameters that can be derived from data (Vestergaard & Hansen, 2006 ▸) through Bayesian indirect Fourier transformation (BIFT) (Hansen, 2000 ▸). It was chosen instead of the number of Shannon channels (Shannon, 1949 ▸; Nyquist, 1928 ▸) as *N*_g,BIFT_ takes into account the noise level of data (Vestergaard & Hansen, 2006 ▸) (see Fig. S1 of the supporting information). *N*_g,BIFT_ was calculated with a BIFT algorithm (Hansen, 2000 ▸), as implemented in *BayesApp* (version 1.1) (Hansen, 2012 ▸; Larsen & Pedersen, 2021 ▸). BIFT cannot fit all data, so one may, in those cases, replace *N*_g,BIFT_ by the number of Shannon channels.

### 2.7. Estimating degrees of freedom to calculate reduced χ^2^ values

The number of good parameters *N*_g_ is a good measure for the degrees of freedom (DOF) in a fit and can therefore provide a correct estimate of the reduced χ^2^, namely DOF = *M* − *N*_g_, where *M* is the number of datapoints (Larsen *et al.*, 2018 ▸; Larsen & Pedersen, 2021 ▸). This is also the case for simultaneous fitting against multiple data (Fig. S2). However, it is not evident what the DOF (and reduced χ^2^ values) should be for each dataset in a simultaneous fit. The number of good parameters for each dataset (*N*_g,*j*_) should add up to the total *N*_g_ for the simultaneous fit. An upper limit of *N*_g,*j*_ can be estimated following the usual approach (Larsen *et al.*, 2018 ▸) for each dataset and is denoted *n*_g,*j*_. The sum of these values is denoted *n*_all_. By requiring that the sum of *N*_g,*j*_ values should equal the total *N*_g_, we reach
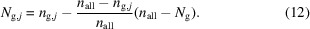
This is a good measure for the DOF, as assessed by monitoring the reduced χ^2^ from simultaneously fitting against simulated data (Fig. S3).

### 2.8. Molecular dynamics simulations

The deposited structure ‘model-1 (pdb)’ (SASBDB entry SASDNK2; Yunoki *et al.*, 2022 ▸) was used as the initial frame. The structure was solvated in TIP3P water with 100 m*M* NaCl, in a cubic box with box lengths of 27 nm and periodic boundary conditions. Simulations were run in *GROMACS 2021.4* (https://www.gromacs.org) with force fields AMBER14SB_OL15 or CHARMM36-IDP. The structure was minimized, equilibrated with a constant number of particles, volume and temperature (NVT) for 100 ps, and then equilibrated with a constant number of particles, pressure and temperature (NPT) for another 100 ps. The protein was position restrained during these equilibration steps, with temperature 300 K and time constant 0.1 ps kept with the *v-rescale* algorithm. The pressure was kept at 1 bar using Parrinello–Rahman pressure coupling and a time constant of 2 ps. The restraints were released and the simulation was run for 100 ns with NPT.

### 2.9. Calculating theoretical scattering from the molecular dynamics simulations

The first 40 ns of the simulations were excluded to avoid the results being dependent on the initial frame. The theoretical scattering was calculated from the remaining 60 ns with *Pepsi-SANS* (for Linux) version 3.0 (https://team.inria.fr/nano-d/software/pepsi-sans) or *Pepsi-SAXS* (version 3.0 for Linux) (Grudinin* et al.*, 2017 ▸). For the SANS data, the scattering from the KaiA domain only was compared with data, as the KaiB and KaiC domains were matched out in the experiment (Yunoki *et al.*, 2022 ▸).

## 3. Results

This section contains two parts. In the first, it is investigated which weighting scheme is best when simultaneously fitting multiple SAXS or SANS contrasts. In the second part, the inclusion of priors is investigated.

### 3.1. Finding the best weighting scheme

When refining a model against multiple datasets, *e.g.* a SAXS and a SANS dataset, or multiple SANS contrasts, a central question is how to weight each dataset. The model refinement is done by minimizing the weighted sum: 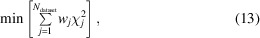
where χ^2^ is defined in equation (9). Assuming independent datapoints, the sum of χ^2^ should be minimized with no additional weighting, *i.e.w*_*j*_ = 1. This naive weighting scheme is the first that will be tested. However, equation (13) is a sum over the non-reduced χ^2^, which scales with the number of datapoints, so the result is dominated by the larger dataset. To counteract this, one may use the weight *w*_*j*_ = 1/*M*_*j*_. This is the second weighting scheme that will be tested. It roughly corresponds to replacing χ^2^ with the reduced χ^2^ in equation (13), so it will be denoted the reduced weighting scheme. A third approach is to weight by the information content in data, *e.g.* by the number of good parameters *N*_g,BIFT_ (Vestergaard & Hansen, 2006 ▸). That way, the data with the highest information content also get the highest weight, *i.e.w*_*j*_ = *N*_g,BIFT,*j*_/*M*_*j*_. A similar information-based weighting scheme has previously been applied to combine SAXS and molecular dynamics simulations (Shevchuk & Hub, 2017 ▸).

In order to test which weighting scheme performs best, two datasets were simulated for a sample of core–multishell particles. The particles had three shells, so a total of four radii were refined from the data. The true values were 10, 30, 50 and 70 Å. The first dataset contained 400 datapoints with a relatively high signal-to-noise ratio, while the second dataset contained only 50 datapoints and a lower signal-to-noise ratio. These data mimic an experiment where the sample is measured with two different contrast situations, *e.g.* with synchrotron SAXS and with SANS (Fig. 1). Most SANS data contain more points than 50 and often there will be multiple SANS contrasts, so the total number of SANS datapoints could often exceed the number of SAXS datapoints. However, the low number was chosen to explore a situation with a substantial difference between the size of the two datasets, *i.e.* where the weight schemes are more important. The true model that was used to generate the simulated data was then refined against the simulated SAXS-like and SANS-like datasets using the three weighting schemes, *w*_*j*_ = 1, *w*_*j*_ = 1/*M*_*j*_ or *w*_*j*_ = *N*_g,BIFT,*j*_/*M*_*j*_, to estimate the geometric parameters and compare with the true values. The model parameters were also refined against SAXS data alone and SANS data alone. To mimic an experiment, the simulated data were generated stochastically. Therefore, the simulation and analysis protocol was repeated 50000 times (*n*_rep_) for each weighting scheme to get a distribution of refined parameter values. The best weighting scheme is the one that gives the most accurate parameter values after refinement, *i.e.* closest to the ground truth. To quantify the accuracy of the determination of each parameter, the deviation from the true value was defined as 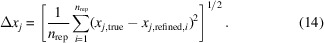
Since the true value is known, there are zero DOF, and the denominator is *n*_rep_ and not *n*_rep_ − 1 as in the standard deviation, where the true value must be estimated as the mean. We use the relative deviations Δ*x*_*j*_/|*x*_*j*,true_| to calculate an average relative deviation of a set of parameters: 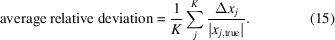


#### 3.1.1. Which weighting scheme is best for refinement of the core–multishell model?

This can be answered by comparing how accurately the structural parameters of the core–multishell model were refined with the different weighting schemes. The radius of the core (*R*_c_) was ill-determined by the data due to its limited size and thus limited scattering contribution [Fig. 1 ▸(*a*)], and due to the low scattering contrast of the core in the SAXS-like data with the highest signal-to-noise ratio. Therefore, it was not uniquely determined using any of the weighting schemes [Fig. 1 ▸(*d*)]. The average relative deviation from the true value, Δ*R*_c_, was 1.7 Å irrespective of the applied weighting scheme, so no weighting scheme was substantially better than the others for this parameter. However, the outer radii of the first and second shells (*R*_1_ and *R*_2_) were refined most accurately when using the naive weighting scheme *w*_*j*_ = 1 (simply using experimental errors as weights), closely followed by the information-based weighting scheme *w*_*j*_ = *N*_g,BIFT,*j*_/*M*_*j*_ (weighting with information content), whereas when using the reduced weighting scheme *w*_*j*_ = 1/*M*_*j*_ (corresponding to using reduced χ^2^ instead of χ^2^), the refined values were substantially less accurate [Figs. 1 ▸(*e*) and 1 ▸(*f*)]. For the outer radius of the third shell (*R*_3_), the naive weighting scheme and the information-based weighting scheme resulted in equally accurate results [Fig. 1 ▸(*g*)].

In order to assess the accuracy of a given weighting scheme using a single number, the average deviation across the radii was calculated, as in equation (15). The average deviation across all radii was 6.4% for the naive weighting scheme, 6.5% for the information-based weighting scheme and 7.8% for the reduced weighting scheme. So the naive weighting scheme performed best for these data as its average deviation was the smallest.

To investigate the generality of the result, other conditions were tested using the same approach, as summarized in Table 1 ▸. This included changing the number of points in each dataset, adding a SANS dataset for highlighting the core radius and adding interparticle interactions. The effect of an inaccurate model and resolution effects were also investigated. This was all done with the spherical core–multishell model (Fig. 1 ▸). Finally, the weighting schemes were evaluated against a stacked-cylinder model (Fig. 2 ▸).

Emphasis was placed on the geometric parameters, namely the radii for the core–multishell model or the lengths and radius for the stacked-cylinder model, as these parameters were co-refined by both sets of data.

#### 3.1.2. Effect of changing the number of points in each dataset

To investigate the effect of the number of points in data, the same spherical core–multishell model was used but new pairs of SAXS- and SANS-like data were simulated with the number of points in the datasets being varied. The ratios of points in the two datasets spanned from 1:1 (300 points in each dataset) to 1:40 (50 and 2000 points, respectively). When the number of points were the same, all weighting schemes performed equally well. However, as the difference in number of points increased, the naive weighting scheme gave the most accurate results (Table 1 ▸). Notably, all weighting schemes were superior to fitting against SAXS or SANS data alone. A substantial difference between the naive weighting scheme and the information-based weighting scheme was observed only when the ratio of points between datasets was at least a factor of 6. On the other hand, the reduced weighting scheme always resulted in less accurate parameter refinement (Table 1 ▸, rows 1–4).

#### 3.1.3. More than two contrasts included

Additional datasets with complementary contrast situations are often measured if the sample contains multiple internal scattering-length densities. Therefore, an additional SANS-like dataset was simulated where only the core had non-zero scattering contrast with respect to the buffer. The spherical core–multishell model was then fitted against the two original datasets (Fig. 1 ▸) and the new SANS dataset that highlights the core. Unsurprisingly, this addition dramatically improved the accuracy of the core radius refinement, *R*_c_ (Fig. S4). However, the conclusions regarding the choice of weighting scheme remained the same; the naive weighting scheme gave the most accurate refinement, especially when there were significant differences between the number of datapoints in each dataset (Table 1 ▸, rows 5 and 6).

#### 3.1.4. Interparticle interactions

If there are interparticle interactions and correlation between the locations of individual particles, a simple form factor is not a sufficient description, and addition of a structure factor is necessary. To investigate this situation, data were simulated with a hard-sphere structure factor to consider interparticle interactions of highly concentrated samples. The same hard-sphere structure factor was used when fitting the data. For the combination of a simulated SAXS dataset with 400 points and a simulated SANS dataset with 50 points, the information-based weighting scheme had the smallest deviation from the true parameter values. However, as the difference in number of points between the datasets increased, the naive weighting scheme gave the smallest average deviation (Table 1 ▸, rows 7 and 8).

#### 3.1.5. Systematic errors: inaccurate models and resolution effects

Examples of systematic errors include interparticle interactions where the structure factor is assumed to be unity, aggregation or oligomerization of a sample that is assumed to be monodisperse, or roughness of surfaces that are modeled as smooth. Systematic errors may also stem from undesired experimental effects, including reflections from the sample holder or buffer mismatches.

To investigate one of these systematic errors, data were simulated using a model with a raspberry-like surface. This model was similar to the core–multishell model, except that the outer shell (shell number 3) was removed and instead the surface of shell number 2 was covered by small spheres. The data were, however, still fitted with the simpler core–multishell model. So the data were simulated with one model but fitted with a simpler inaccurate model. This resulted in large variation of the refined values (Table 1 ▸, rows 9 and 10) due to ambiguous determinations of the outer two shells (Fig. S5). However, despite the inaccurate model, the naive weighting scheme remained the most accurate (Table 1 ▸, rows 9 and 10).

Resolution effects are another important aspect to consider, especially in SANS. As neighboring points are related through smearing effects, one may suspect that the naive weighting scheme, which assumes independent datapoints, would perform worse. Therefore, resolution effects were applied to the simulated SANS data and were likewise included in the subsequent fitting process. These effects, which are described as an uncertainty in *q*, were multiplied by factors of 2 or 3 to simulate more severe resolution effects. In all cases, however, the naive weighting scheme outperformed the other weighting schemes (Table 1 ▸, rows 11–16).

#### 3.1.6. Changing the model: stacked cylinders

To challenge the generality of the results, a cylinder model was tested. This model consisted of three cylinders stacked along the longitudinal axis. Each cylinder had the same radius but the cylinder lengths and scattering-length densities varied (Fig. 2 ▸). This model was less symmetric than the core–shell model and represented a different contrast situation. How­ever, the conclusion remained the same: the naive weighting scheme provided the most accurate results, followed by the information-based weighting scheme, and both were much better than the reduced weighting scheme (Table 1 ▸). Notably, when fitting against simulated SAXS data with 2000 points and simulated SANS data with 50 points, only the naive weighting scheme was superior to refinement against SAXS data alone. For the two other weighting schemes, the refined parameters became less accurate from inclusion of an additional SANS dataset with different contrast but much fewer points (Table 1 ▸, bottom two rows).

### 3.2. Effect of over- or under-estimated errors

To investigate the effect of poor error estimates, data were simulated again using the core–multishell model, but this time the errors of either the SANS or the SAXS data were multiplied with a factor between 0.1 and 10 after they had been simulated. Thus, the reported errors of the simulated data no longer reflected the fluctuations of the data around the true value. The errors ranged from highly underestimated (a factor of 0.1) to highly overestimated (a factor of 10).

**Figure 3:**
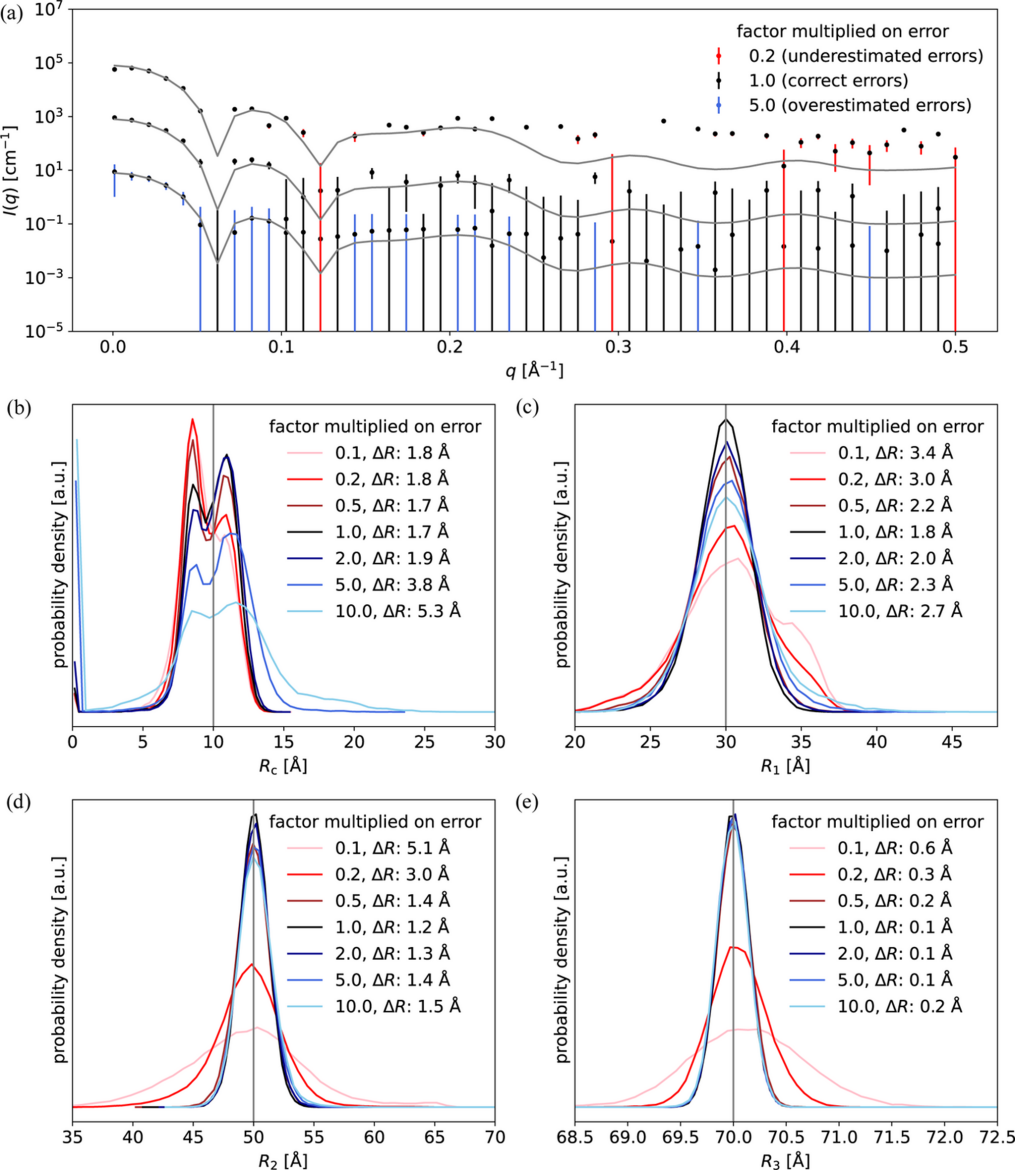
Effect of over- or under-estimated errors on parameter refinement. (*a*) Examples of simulated SANS data with over- or under-estimated errors. (*b*)–(*e*) Radii of the core–multishell model when refined 50000 times against SAXS and SANS data, with the latter having errors that are over- or under-estimated by a factor between 0.1 (highly underestimated) and 10 (highly overestimated).

In the first round, the SAXS data were kept unchanged while the SANS errors were changed to be either underestimated or overestimated. The radii of the core–multishell model were then estimated against the SAXS and altered SANS data. Not surprisingly, the radii were determined most accurately when the errors were correct (Fig. 3 ▸). Overestimation of the SANS errors had severe effects on the core radius in the core–multishell model (*R*_c_) because this parameter was predominantly determined by the SANS data. On the other hand, underestimation of the SANS errors had little effect on *R*_c_ but made the estimation of the outermost radius *R*_2_ worse, as this parameter was predominantly determined from the SAXS data, and SANS errors that were too low effectively gave too little weight to the SAXS data (Fig. 3 ▸). In the second iteration, the roles were shifted and the errors in the SAXS data were varied, while keeping the SANS errors at the correct level (Fig. S6). In this case, the most severe effects were observed for *R*_c_ when the SAXS errors were underestimated. These results illustrate that over- or under-estimation of errors can lead to poorer estimates of the refined model parameters. The effect depends on the contrast situation, the signal-to-noise ratio of the datasets, and the degree of over- or under-estimation. Therefore, errors should be assessed and, if possible, corrected before model refinement against multiple SAXS/SANS datasets (Larsen & Pedersen, 2021 ▸; Smales & Pauw, 2021 ▸).

### 3.3. Inclusion of priors

Now we turn our focus towards how prior information can be included in the modeling. In conventional model refinement, no prior distribution is explicitly attributed to the parameters, but most fitting programs allow the user to set a minimum and a maximum value for each parameter (Kohlbrecher & Breßler, 2022 ▸; Ilavsky & Jemian, 2009 ▸). This is equivalent to applying a uniform distribution for each parameter. So far in this paper, we have used such uniform priors, only limiting the parameters to a certain range around the true value and preventing negative values where relevant. The simplest alternative is Gaussian priors, which are defined by a mean μ_prior_ and a standard deviation σ_prior_. Gaussian priors can be included using Bayesian refinement. It has previously been shown that inclusion of Gaussian priors (as opposed to uniform priors) improves the robustness of the refinement (Larsen *et al.*, 2018 ▸). However, this was only shown for the refinement against a single SAXS/SANS dataset. Multiple datasets can be fitted simultaneously by minimizing the sum 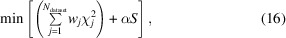
where *S* represents the prior and α is the effective weight given to the prior. To investigate the effect of the prior, the naive weighting scheme was used on simulated data of core–multishell particles. The model parameters were co-refined against a SAXS-like dataset with 400 points and a SANS-like dataset with 50 points.

#### 3.3.1. Description of prior distributions

Three sets of Gaussian prior distributions were generated (‘poor prior’, ‘good prior’ and ‘best prior’), where the best prior is the set of priors that are closest to the true values. The Gaussian priors were truncated, with the minimum and maximum values defined as being five standard deviations from the mean (μ ± 5σ). For the radii, a lower limit of 0 was also set if μ − 5σ < 0. A non-informative uniform prior was generated for comparative analysis (Uniform_5σ_), which was constant between the upper and lower limits and zero outside this interval.

Prior values for the radii are given in Table 2 ▸. All priors had the same values for all other parameters, *i.e.* scattering contrasts, scaling and background (Table 3 ▸).

#### 3.3.2. Gaussian priors improve the accuracy of the refined parameters

**Figure 4:**
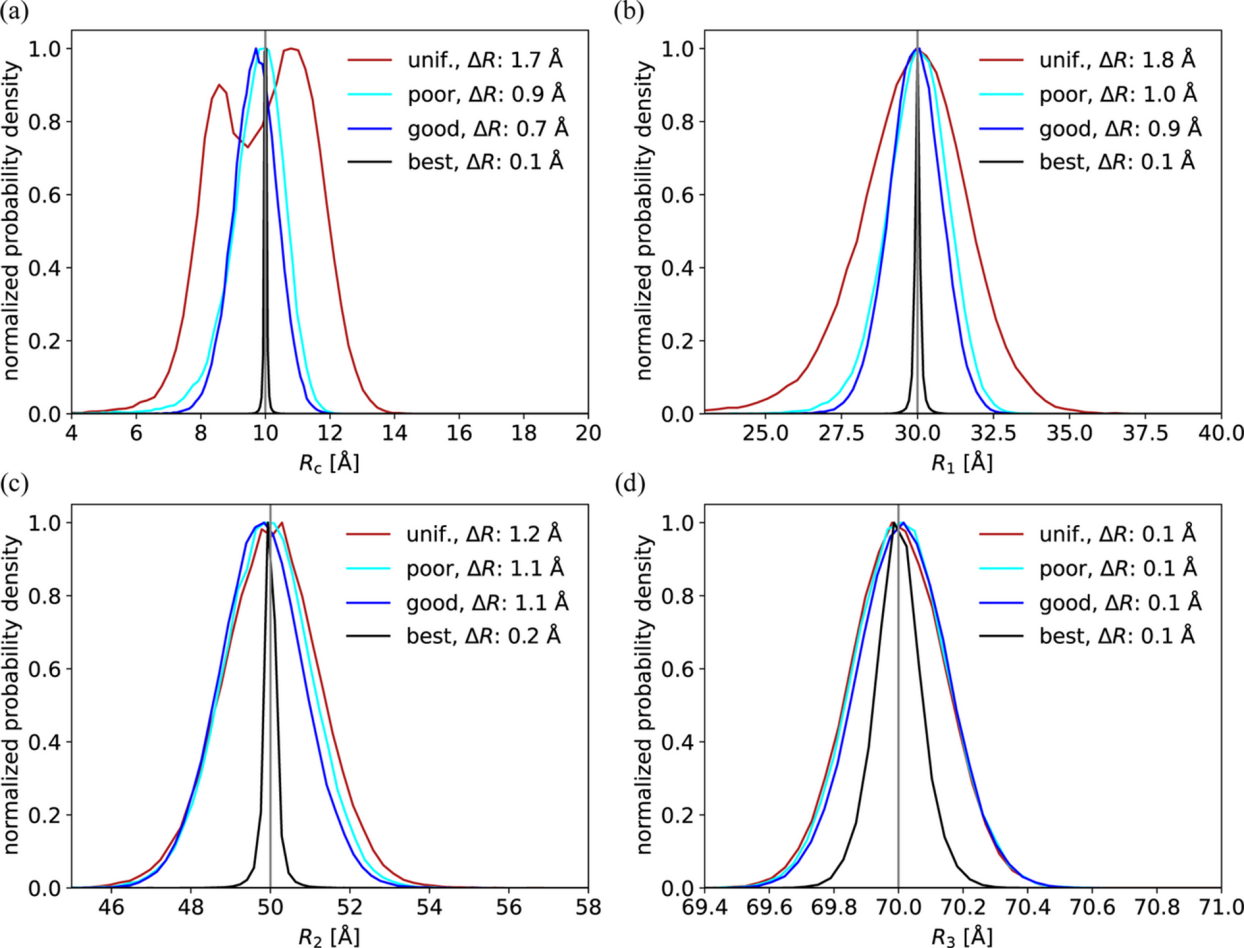
Radii of the core–multishell model were refined against SAXS and SANS data using a non-informative uniform prior (red), a poor Gaussian prior (light blue), a good Gaussian prior (dark blue) or the best Gaussian prior (black). The probability distributions were normalized, such that their maximum value is unity.

The estimates of *R*_c_, *R*_1_ and *R*_2_ were substantially improved by all tested Gaussian priors compared with the non-informative uniform prior (Fig. 4 ▸). The best prior resulted in a very narrow distribution of refined values, although the prior width was relatively wide (Fig. S7 and Table 2 ▸). The refinement of *R*_3_, on the other hand, was not improved by inclusion of Gaussian priors, as this parameter is very well defined by the data. Generally, the better a parameter was determined from data itself, the smaller the effect of the prior. Importantly, the priors did not worsen the refined parameter values, even when the priors were relatively poor (Fig. 4 ▸).

#### 3.3.3. Improving the uniform priors

The uniform prior was stepwise improved by narrowing the upper and lower bounds from μ_best_ ± 5σ_best_ to μ_best_ ± (1/2)σ_best_, where μ_best_ and σ_best_ are the mean and standard deviation of the best Gaussian prior. The results got increasingly more accurate, but even the narrowest uniform prior gave substantially larger deviations than the best Gaussian prior (Fig. S8). Remarkably, the poor prior resulted in a smaller deviation compared with all uniform priors with minimum and maximum values of ±1σ_best_ or higher. This illustrates that Gaussian priors are better than uniform priors at guiding the minimization algorithm towards the correct solution, while maintaining a larger prior solution space.

## 4. Experimental example: circadian clock protein complex

In an elegant study by Yunoki *et al.* (2022 ▸), the structure of the circadian clock protein complex was determined with SAXS and SANS. The protein complex has a sixfold symmetry and contains six identical subunits. Each subunit consists of multiple domains, called KaiA, KaiB and KaiC. In the SANS experiment, the KaiB and KaiC domains were matched out. SAXS and SANS data were thus complementary and could exclude different structural candidates; in particular, the SANS data excluded two proposed structure classes (Type 2 and Type 3) (Yunoki *et al.*, 2022 ▸). The data were deposited in the SASBDB with IDs SASDNK2 (SANS data) and SASDNJ2 (SAXS data).

Here, the data were used to showcase the use of priors and weights in simultaneous fitting of multiple SAS datasets. First, the experimental errors were assessed using the BIFT algorithm (Larsen & Pedersen, 2021 ▸). The SANS errors were assessed to be correct, whereas the SAXS errors were assessed to be slightly underestimated, so these were rescaled by a factor of 1.6 to obtain a better balance between SAXS and SANS data.

**Figure 5:**
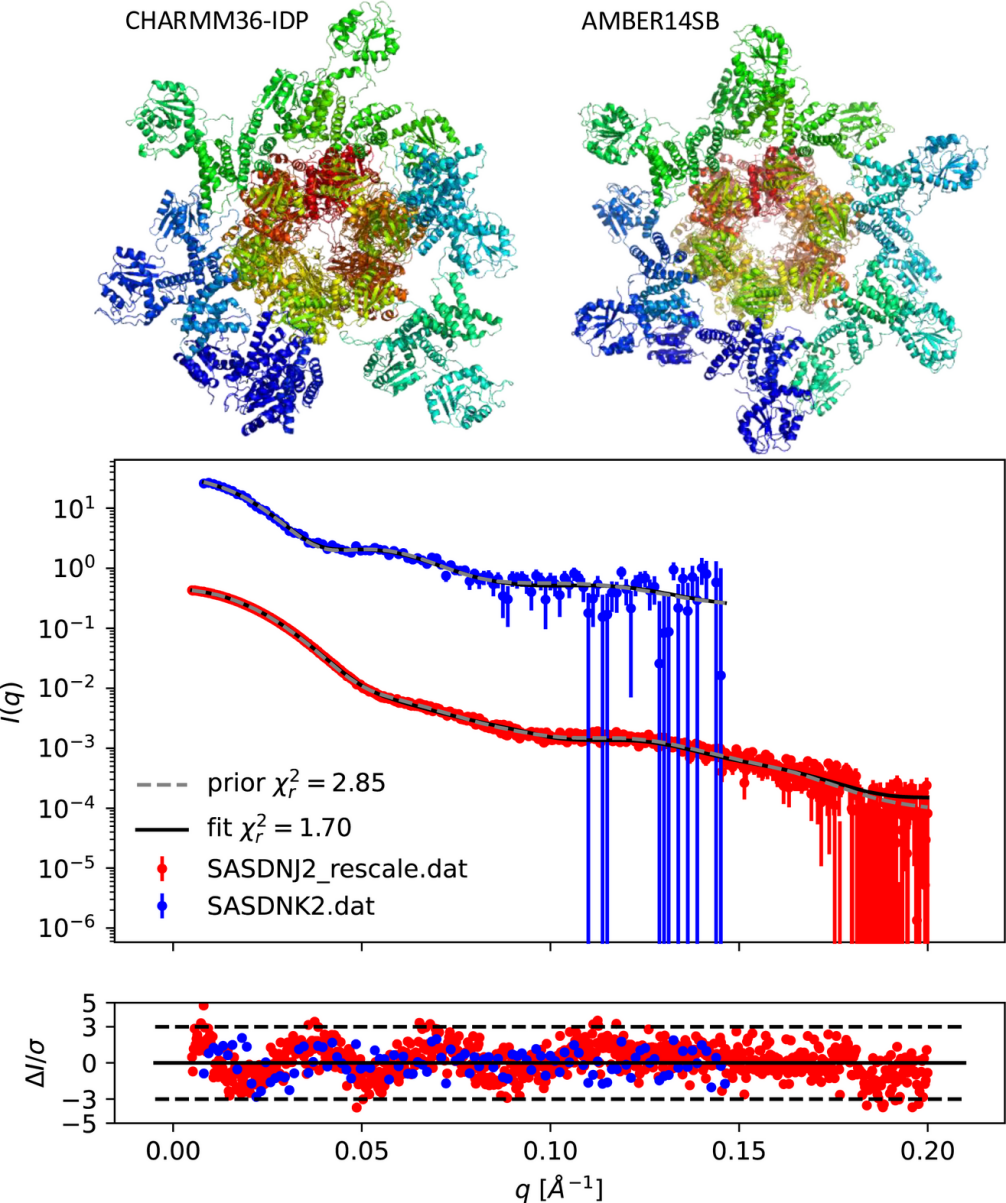
(*a*) Representative snapshots from the two simulated ensembles, with the CHARMM36-IDP force field leading to asymmetric structures and the AMBER14SB force field leading to symmetric structures. The central part of the protein complex was matched out in SANS (KaiB and KaiC) (Yunoki *et al.*, 2022 ▸). (*b*) Simultaneous fitting of SAXS (with rescaled errors, SASDNJ2, red) and SANS data (SASDNK2, blue), displaying also the prior, with equal amounts of the two structural ensembles. Normalized residuals displayed below the fits.

A model structure deposited at the SASBDB entry SASDNJ2 was used as the initial structure, and a 100 ns simulation was run with two different force fields to probe various structural arrangements and their consistency with the SAXS and SANS data. The first force field, AMBER14SB, provides an ensemble of relatively symmetric structures, whereas the second force field, CHARMM36-IDP, was developed for intrinsically disordered proteins and breaks the symmetry of the complex (Fig. 5 ▸). The symmetric structural ensemble generated with the AMBER14SB force field was consistent with the data, with a reduced χ^2^ (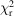) value of 1.7 for the simultaneous fit. The asymmetric structural ensemble generated with the CHARMM36-IDP force field was less consistent with the data (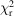 = 6.6). However, there could be a minor fraction of asymmetric structures in the sample, as observed for other protein multimers (Johansen *et al.*, 2022 ▸). To determine whether this was the case for the circadian clock protein complex, a mixture of the structural ensemble was used to fit the data, where *f*_sym_ and *f*_asym_ are the fraction of structures from the symmetric (AMBER14SB) and asymmetric (CHARMM36-IDP) ensembles, respectively, and the calculated scattering from each ensemble is *I*_sym_ and *I*_asym_, respectively. The mixed scattering can then be described as 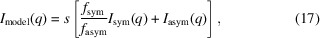
where *s* is an overall scaling parameter. A non-informative log-normal prior distribution was used for the stoichiometric ratio, 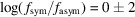, corresponding to assuming that half of the ensemble structures are symmetric and half are asymmetric.

By simultaneous fitting of the SAXS and SANS data using the naive weighting scheme, the stoichiometry was refined to 90% [88, 91] (68% confidence interval) symmetric structures from the AMBER force field ensemble and 10% [9, 12] asymmetric structures from the CHARMM36-IDP force field ensemble, with a 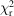 of 1.7 for the total simultaneous fit. Furthermore, 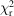 was 1.8 for the simultaneous fit against the SAXS data and 1.2 for the fit to SANS.

Using the reduced weight scheme, the stoichiometry was instead refined to 89% [15, 98] symmetric and 11% [2, 85] asymmetric with the same goodness of fit as above, but with much higher uncertainty on the refined parameters. Refining against SAXS data alone gave the same result as the naive weighting scheme, whereas refinement against SANS alone gave 77% [57, 89] symmetric and 23% [11, 43] asymmetric structures. If the SAXS errors were not rescaled, the resulting stoichiometry (and confidence interval) was, in this case, essentially unchanged, but with a larger 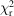 of 4.1 for the fit (4.5 for SAXS and 1.2 for SANS).

Overall, in this example, SAXS is dominating in discriminating between the two structural ensembles. But this was not obvious, and using optimal weighting ensures that the most accurate solution is robustly found. The structural conclusion is that the addition of the asymmetric structure does not improve the fit to data compared with only using the symmetric ensemble, which supports the modeling strategy taken by Yunoki *et al.* (2022 ▸), namely using the AMBER14SB force field.

## 5. Discussion

### 5.1. Choice of tested models

Two models were tested: a core–multishell model and a model of stacked cylinders. The motivation was to cover models with different shape and symmetry, yet possessing a complex geometry, where multiple parameters should be co-refined against data. This was supplemented with additional tests, *e.g.* examining structure factors and resolution effects, leading to the same conclusion. Other aspects and models have not been tested, such as inclusion of polydispersity or rough interfaces. However, there is no reason that these effects should lead to different conclusions, as long as they can be modeled with a set of model parameters, *e.g.* through a size distribution in the case of polydispersity or as Gaussian smearing in the case of interface roughness.

### 5.2. Why is the model refinement not dominated by the dataset with many datapoints?

Even when one dataset had 2000 datapoints and the other only 50 datapoints, the refined parameters were still affected by both datasets. This is because the data contained orthogonal information. For some structural domains, the scattering contrast was low in SAXS and high in SANS. Therefore, an additional dataset can contain much structural information, despite having a low signal-to-noise ratio. On the other hand, if the contrast situation is similar in multiple SAS datasets that are simultaneously fitted, then the refined parameters will be dominated by the dataset with the better signal-to-noise ratio (Pedersen *et al.*, 2014 ▸; Larsen *et al.*, 2020 ▸; Larsen & Pedersen, 2021 ▸).

When datapoints are statistically independent, no additional weighting is necessary, *i.e.* the naive weighting scheme leads to the most accurate result, as demonstrated with the simulated data. Oversampling of data, *i.e.* the number of datapoints exceeds the number of Shannon channels, which was the case for the simulated data, does not lead to statistical dependency. However, it is crucial to avoid operations in the data reduction process that introduce dependence, or to take these operations into account in the error propagation (Heybrock *et al.*, 2023 ▸).

### 5.3. When experimental errors are ill-defined

Error estimates are important for getting the correct balance between multiple datasets when carrying out model co-refinement (Fig. 3 ▸). Methods have previously been presented to identify, and in some cases correct, over- or under-estimated errors (Larsen & Pedersen, 2021 ▸; Smales & Pauw, 2021 ▸). However, there is only limited work on how to identify systematic errors, *e.g.* from non-optimal buffer subtraction (Shevchuk & Hub, 2017 ▸). This becomes particularly important when high flux, long exposure times and stable samples at high concentrations allow the statistical errors to reach a level where the fluctuations in data are dominated by errors that are not accounted for. Such effects may likely be the cause of why the SAXS dataset used in the experimental example (SASBDB entry SASDNJ2) was assessed to have underestimated errors by the BIFT algorithm. Goodness-of-fit measures that exploit runs tests do not depend on statistical errors and are therefore valuable tools for identifying variations that are not reflected in the counting-statistics-based errors (Franke *et al.*, 2015 ▸; Koefinger *et al.*, 2021 ▸).

## 6. Conclusions

The most optimal weighting scheme for simultaneous fitting of multiple datasets is simply *w*_*j*_ = 1. That is, the sum of the (non-reduced) χ^2^ values should be minimized. This was compared with a weighting scheme with the information content taken into account (*w*_*j*_ = *N*_g,BIFT,*j*_/*M*_*j*_) and with a weighting scheme relying on reduced χ^2^ values rather than χ^2^ values (*w*_*j*_ = 1/*M*_*j*_). The naive weighting scheme (*w*_*j*_ = 1) gave the most accurate results, in particular when there was a substantial difference in the number of points in each included dataset.

Inclusion of Gaussian priors gave more accurate refinement of structural parameters than using uniform priors. This has previously been demonstrated for single SAXS datasets (Larsen *et al.*, 2018 ▸), but here it was demonstrated that this was also the case when simultaneously fitting multiple SAXS or SANS datasets.

Implementing optimal strategies for data analysis, as proposed in this study, is a pragmatic approach to enhance the accuracy of structural refinements. These strategies require minimal resources compared with the immense work that is needed to prepare samples and to build and maintain SAXS and SANS instruments. They offer substantial improvement in the accuracy of the refined parameters, and ultimately aid scientists in reaching more accurate and consistent conclusions.

## Supplementary Material

Supporting figures. DOI: 10.1107/S1600576725002390/jl5103sup1.pdf

## Figures and Tables

**Table 1:** Average relative deviation of each weighting scheme for all conditions described in the main text (lower deviation is better), calculated as in equation (15) For the core–multishell model, the structural parameters *R*_c_, *R*_1_, *R*_2_ and *R*_3_ were included in the deviation metric, but nuisance parameters like scaling, background and contrasts were not. For the raspberry model, the core radius and the thickness of the first two layers were considered. For the stacked-cylinder model, the structural parameters *R*, *L*_1_, *L*_2_ and *L*_3_ were included in the deviation measure. Using bootstrapping, the 99% confidence intervals were determined to be ∼1% across the different test cases, which is reflected in the number of significant digits displayed in the table. *M*_N_ and *M*_X_ are the number of points in the simulated SANS-like and SAXS-like datasets, respectively.

	*M*_N_:*M*_X_	*w*_*j*_ = 1	*w*_*j*_ = 1/*M*_*j*_	*w*_*j*_ = *N*_g,BIFT,*j*_/*M*_*j*_	SANS	SAXS
Core–multishell model						
SAXS + SANS	300:300	4.8	4.8†	4.8	8.1	20.4
SAXS + SANS	50:400	6.4	7.3	6.5	12.5	19.4
SAXS + SANS	50:900	6.2	7.8	6.6	12.7	21.5
SAXS + SANS	50:2000	4.7	7.1	5.8	12.6	14.3
Add core contrast	50:400‡	2.1	2.8	2.2	12.8	27.7
Add core contrast	50:2000‡	1.2	2.7	1.8	12.8	15.3
Add structure factor	50:400	12.4	13.0	12.3	18.9	24.1
Add structure factor	50:2000	9.6	12.7	10.8	18.9	19.6
Raspberry-like surface	50:400	50	55	52	77	65
Raspberry-like surface	50:2000	45	52	50	69	60
SANS res. eff. (×1.0)	50:400	6.5	7.3	6.5	13.0	19.6
SANS res. eff. (×1.0)	50:2000	4.9	7.6	6.2	13.3	15.3
SANS res. eff. (×2.0)	50:400	6.8	7.8	7.0	13.3	19.4
SANS res. eff. (×2.0)	50:2000	5.1	8.9	7.2	13.8	15.5
SANS res. eff. (×3.0)	50:400	8.3	9.3	8.4	13.9	19.5
SANS res. eff. (×3.0)	50:2000	6.1	10.8	9.9	14.1	15.4

Stacked cylinder model						
SAXS + SANS	50:400	10.2	12.0	11.6	19.3	25.5
SAXS + SANS	50:2000	5.1	10.4	9.8	18.0	8.0

† When the number of points in the SAXS and SANS datasets are the same, then *w*_*j*_ = 1/*M*_*j*_ is equivalent to *w*_*j*_ = 1.

‡ The additional SANS-like dataset for the core contained 50 points.

**Table 2:** True values and prior values for the radii of the core–multishell model For the Gaussian priors, the mean (μ) and standard deviation (σ) are given along with the upper and lower limits, which are μ ± 5σ, or zero for the lower limit. For the uniform priors, the mean values, μ, were used as the initial value in the fit. For Uniform_5σ_, the minimum and maximum values were the same as for the Gaussian priors, namely μ ± 5σ. The other uniform priors are narrower, with subscripts indicating the distance from μ to the upper/lower limits.

Prior name	*R*_c_ (min,max) (Å)	*R*_1_ (min,max) (Å)	*R*_2_ (min,max) (Å)	*R*_3_ (min,max) (Å)
True value	10	30	50	70
Uniform_5σ_	10 (0, 35)	30 (0, 80)	50 (0, 125)	70 (0, 170)
Uniform_4σ_	10 (0, 30)	30 (0, 70)	50 (0, 110)	70 (0, 150)
Uniform_3σ_	10 (0, 25)	30 (0, 60)	50 (5, 95)	70 (10, 130)
Uniform_2σ_	10 (0, 20)	30 (10, 50)	50 (20, 80)	70 (30, 110)
Uniform_1σ_	10 (5, 15)	30 (20, 40)	50 (35, 65)	70 (50, 90)
Uniform_(1/2)σ_	10 (7.5, 12.5)	30 (25, 35)	50 (40, 55)	70 (60, 80)
Gaussian_poor_	5 ± 5 (0, 30)	40 ± 10 (0, 90)	45 ± 15 (0, 120)	90 ± 20 (0, 190)
Gaussian_good_	8 ± 4 (0, 28)	35 ± 10 (0, 85)	40 ± 20 (0, 140)	80 ± 10 (30, 130)
Gaussian_best_	10 ± 5 (0, 35)	30 ± 10 (0, 80)	50 ± 15 (0, 125)	70 ± 20 (0, 170)

**Table 3:** True values and prior values for all model parameters except radii, which are given in Table 2 ▸ The same means (μ) and standard deviations (σ) were used in all Gaussian priors. For the uniform priors, the means were used as initial guesses. In all priors, uniform and Gaussian, the upper and lower limits were μ ± 5σ.

Parameter	True value	μ ± σ	(μ − 5σ, μ + 5σ)
(Δρ_1_/Δρ_c_)_SAXS_	2	2.0 ± 0.2	(1.0, 3.0)
(Δρ_1_/Δρ_c_)_SANS_	−0.1	−0.10 ± 0.01	(−0.15, −0.05)
(Δρ_2_/Δρ_c_)_SAXS_	3	3 ± 3	(1.5, 4.5)
(Δρ_2_/Δρ_c_)_SANS_	0.1	0.10 ± 0.01	(0.05, 0.15)
(Δρ_3_/Δρ_c_)_SAXS_	4	4.0 ± 0.4	(2.0, 6.0)
(Δρ_3_/Δρ_c_)_SANS_	0.05	0.050 ± 0.005	(0.025, 0.075)
*a*_SAXS_ (cm^−1^)	0.5	0.50 ± 0.05	(0.25, 0.75)
*a*_SANS_ (cm^−1^)	0.8	0.80 ± 0.08	(0.1, 0.9)
*b*_SAXS_ (10^−4^ cm^−1^)	0.1	0.1 ± 100	(−500, 500)
*b*_SANS_ (10^−4^ cm^−1^)	1.0	1.0 ± 100	(−499, 501)
