# Supplementary material for: Optimal weights and priors in simultaneous fitting of multiple small-angle scattering datasets
Source: J Appl Crystallogr. 2025 May 2;58(Pt 3):934–47. doi: 10.1107/S1600576725002390 (PMC12135988; doi:10.1107/S1600576725002390)
Supplement: Supplementary file 1 [file j-58-00934-sup1.pdf]

JOURNAL OF APPLIED CRYSTALLOGRAPHY

Volume 58 (2025)

Supporting information for article:

Optimal weights and priors in simultaneous fitting of multiple small-angle scattering datasets

Andreas Haahr Larsen

Supporting Information
Optimal weights and priors in simultaneous fitting of multiple small-angle scattering datasets

Andreas Haahr Larsen [a]*

[a] *University of Copenhagen, Department of Neuroscience, Blegdamsvej 3, 2200 Copenhagen, Denmark. E-mail: andreas.larsen@sund.ku.dk*

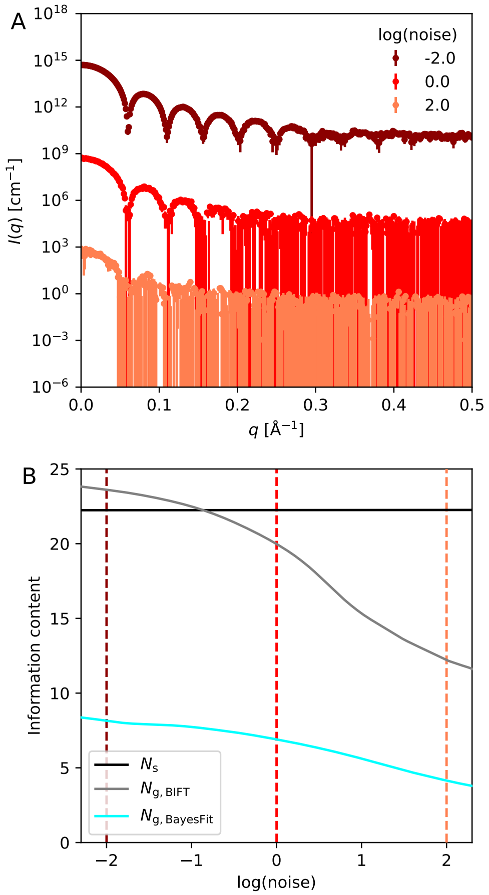

Fig. S1. Information content of simulated data with increasing noise. (A) Examples of data with increasing noise, corresponding to the dashed lines in panel B. (B) The information content as estimated by the number of Shannon channels N_s (black full line) and the number of good parameters $N_{g,BIFT}$ (dark blue) from an indirect Fourier transformation. The number of good parameters were also estimated from model fitting $N_{g,BayesFit}$ (light blue), which converged towards the number of model parameters for low noise and towards zero for high noise levels.

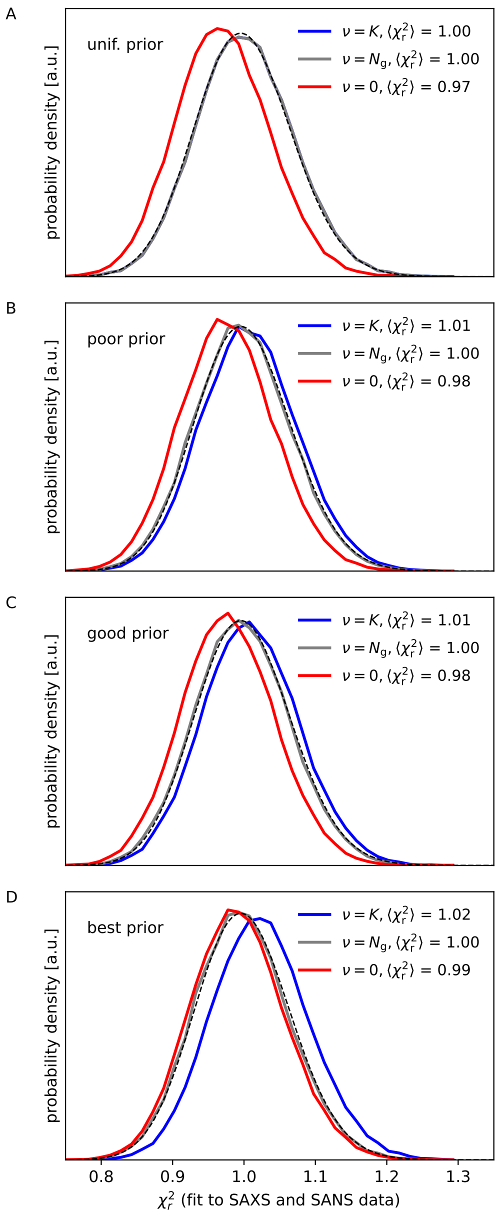

Fig. S2. Distribution of $\chi^2_r = \chi^2/(M - \nu)$ from a simultaneous fit against SAXS and SANS data, using different values for the degrees of freedom: $M - K$ (blue), $M - N_{g,\mathrm{BayesFit}}$ (gray) or M (red), plotted with the theoretical distribution (dashed black). Refinement was done using either the non-informative uniform prior (A-B), the poor Gaussian prior (C-D), the good Gaussian prior (E-F), or the best Gaussian prior (G-H), as described in the main text. $M - N_{g,\mathrm{BayesFit}}$ is the best measure for the degrees of freedom as it aligns with the theoretical distribution.

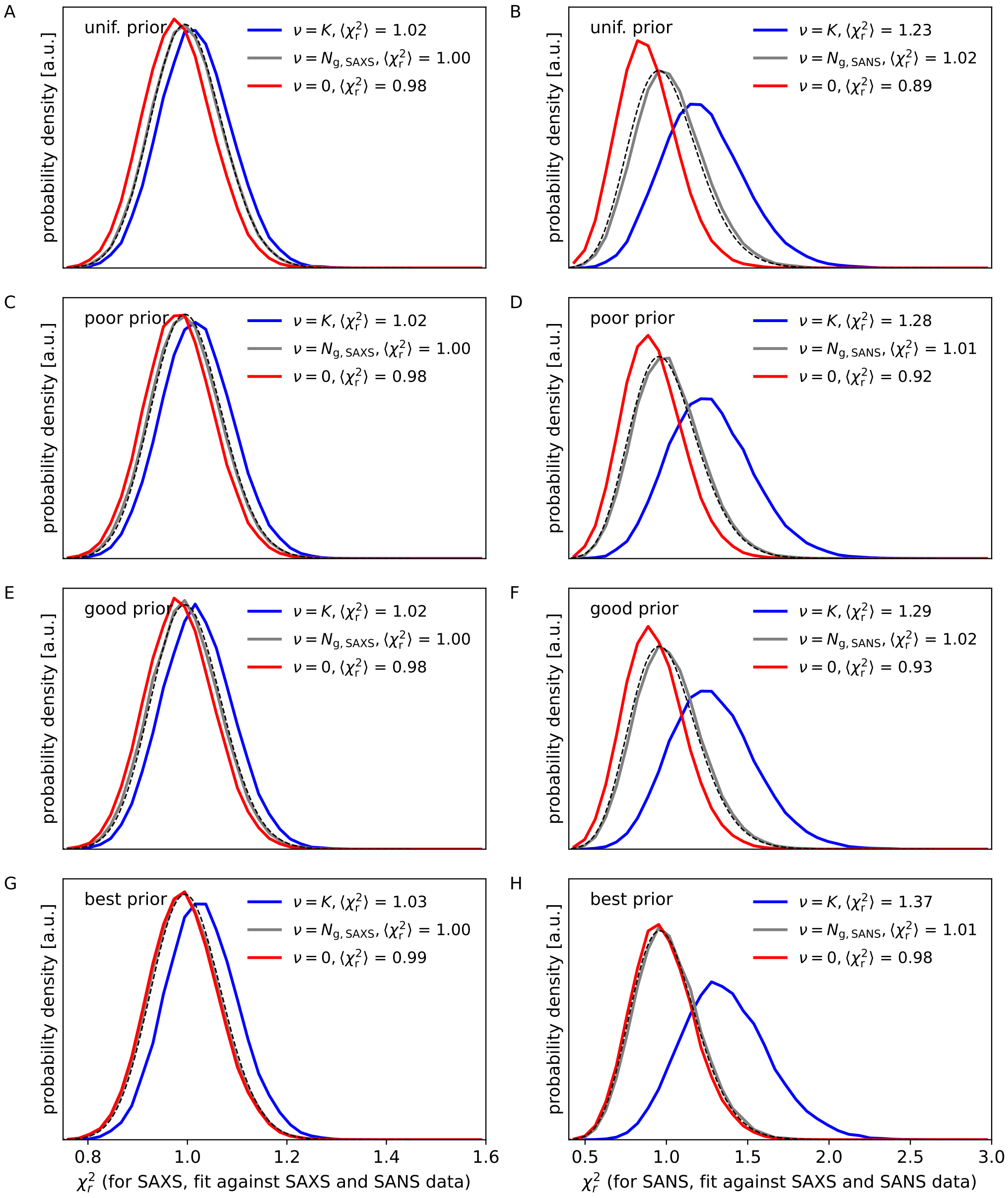

Fig. S3. Distribution of $\chi^2_{\rm r} = \chi^2/(M-\nu)$ for SAXS-like data (left, 400 data points) or SANS-like data (right, 50 data points) after refinement against both data sets. Different values for the degrees of freedom were used: $M-K$ (blue), $M-N_{\rm g,BayesFit}$ (gray) or M (red). Refinement was done using either the non-informative uniform prior (A-B), the poor Gaussian prior (C-D), the good Gaussian prior (E-F), or the best Gaussian prior (G-H), as described in the main text. The effect is most prominent when there are few points in the data, in this case for the simulated SANS data (right). When $M-K$ (blue) was used as the degree of freedom, $\chi^2_{\rm r}$ became too high, but if M (red) was used instead, $\chi^2_{\rm r}$ became too low. Using $N_{g,j}$ provided a good estimate, close to the theoretical value.

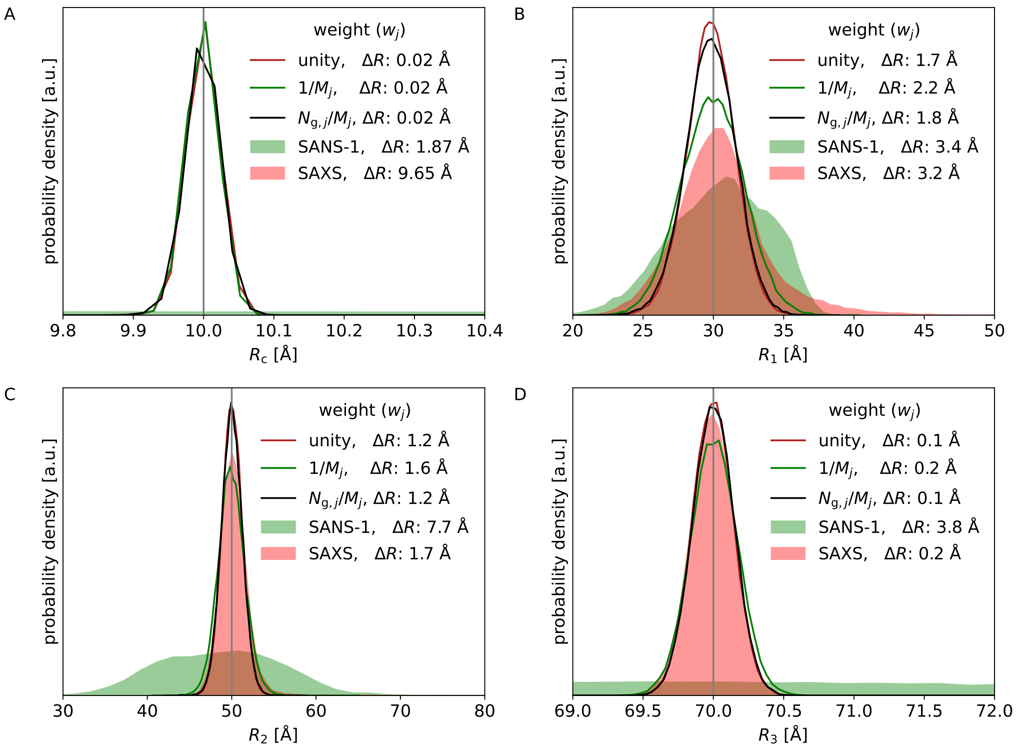

Fig. S4. Radii of the core-multishell model refined against SAXS and two SANS datasets, with one of the SANS datasets matching out all shells, so only the core is left. The refinement of R_c is more accurate after adding the core SANS contrast.

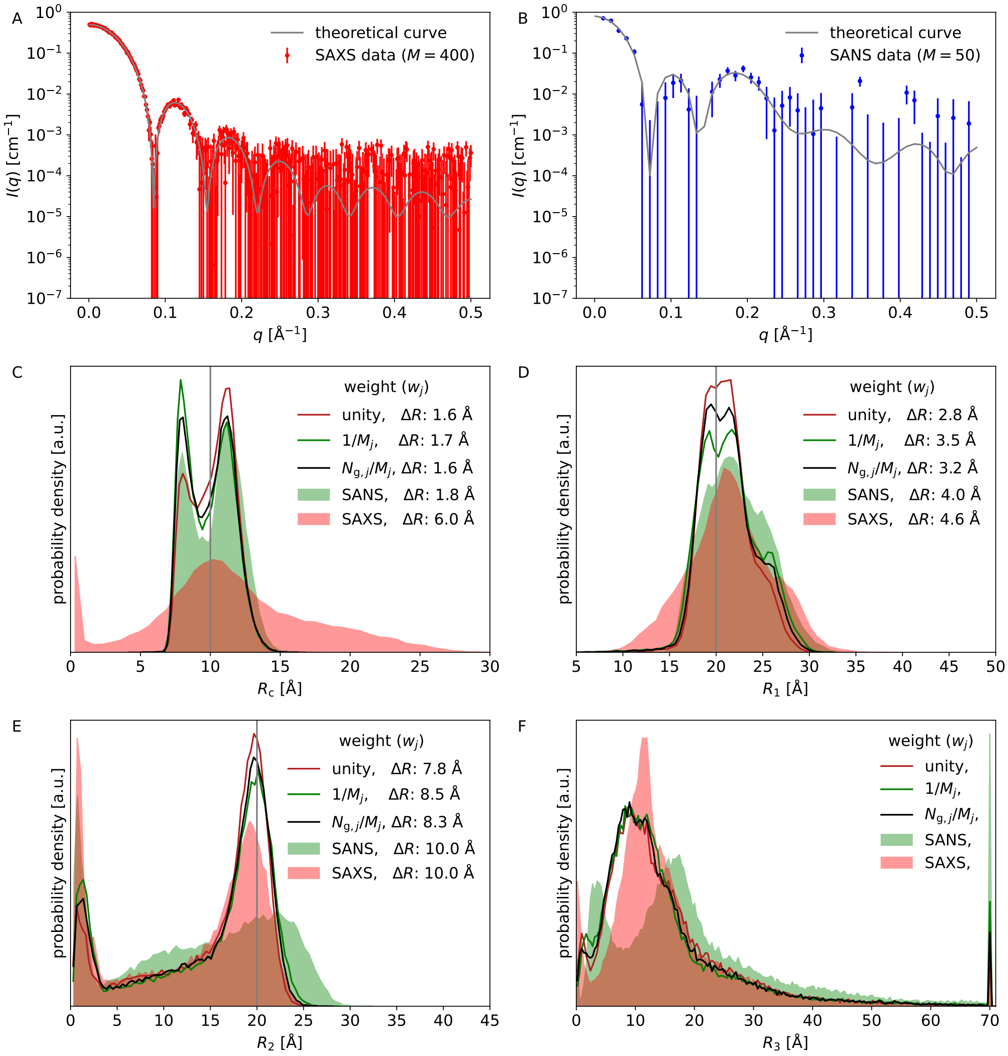

Fig. S5. (A-B) Simulated SAXS and SANS data from a particle consisting of a spherical core and two shells, decorated with small spheres on the surface. Data were fitted with a core-multishell model with core radius R_c (C) and shell radii R_1, R_2 and R_3 (D-F).

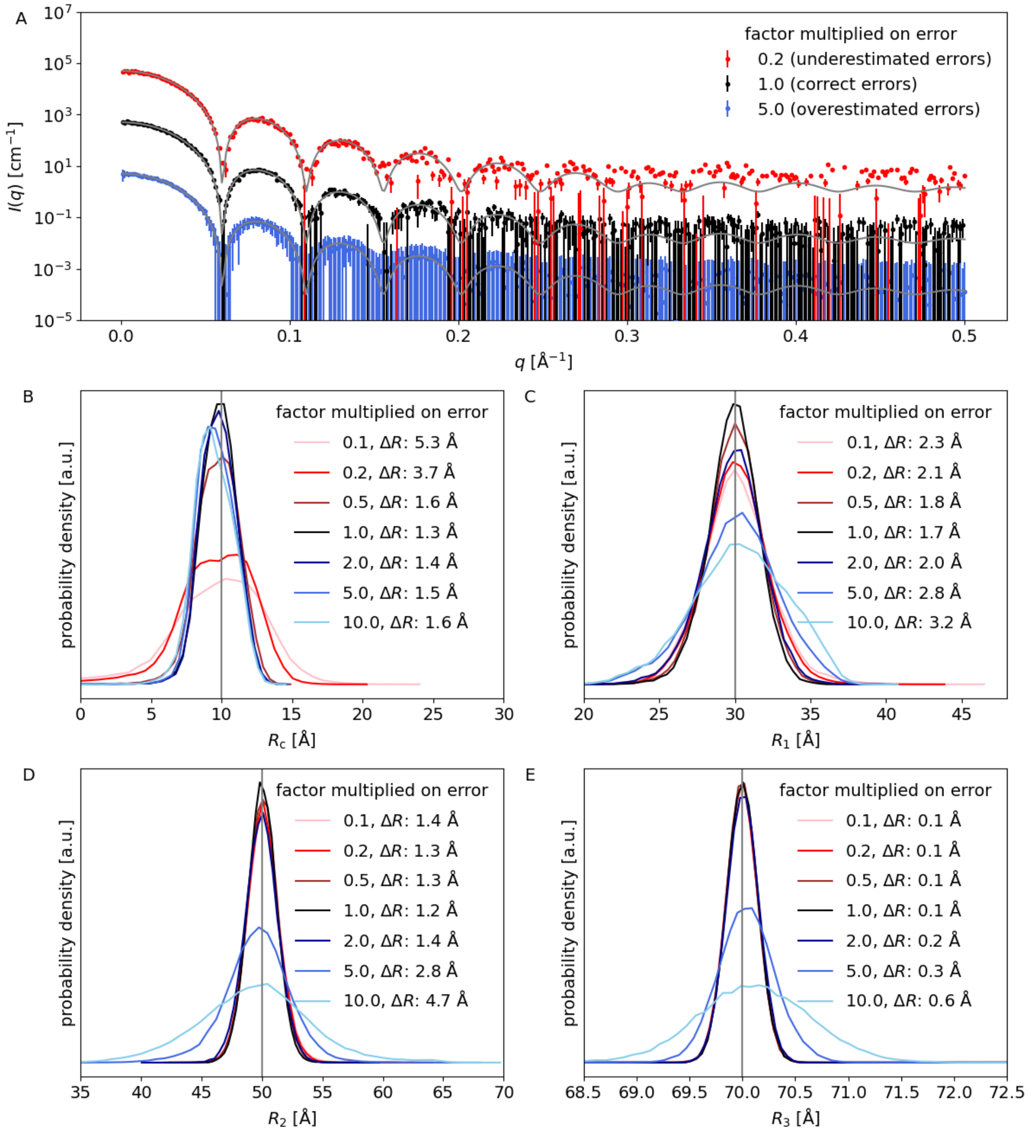

Fig. S6. (A) Simulated SAXS data of core-multishell particle with correct or overestimated or underestimated errors. (B-E) Refinement of the core radius R_c and shell radii against SAXS data with correct (black) or overestimated (blue) or underestimated (red) errors.

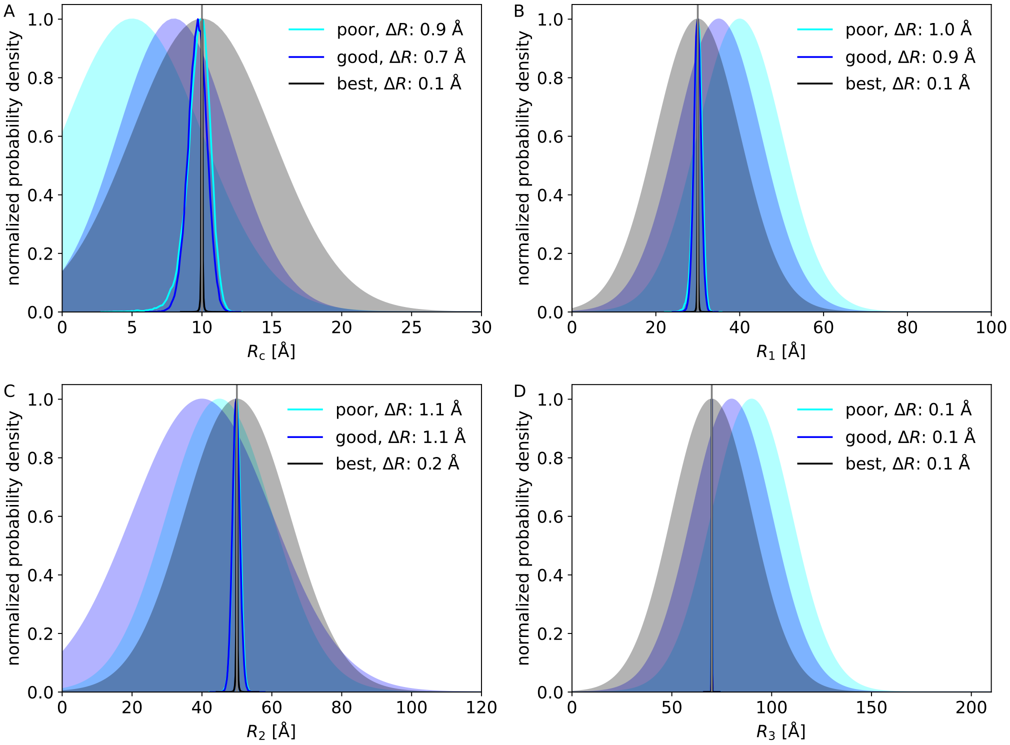

Fig. S7. Radii of the core-multishell model were refined against SAXS and SANS data, using different priors. The priors (filled areas) are plotted along with the distributions of the refined values of the radii (full lines).

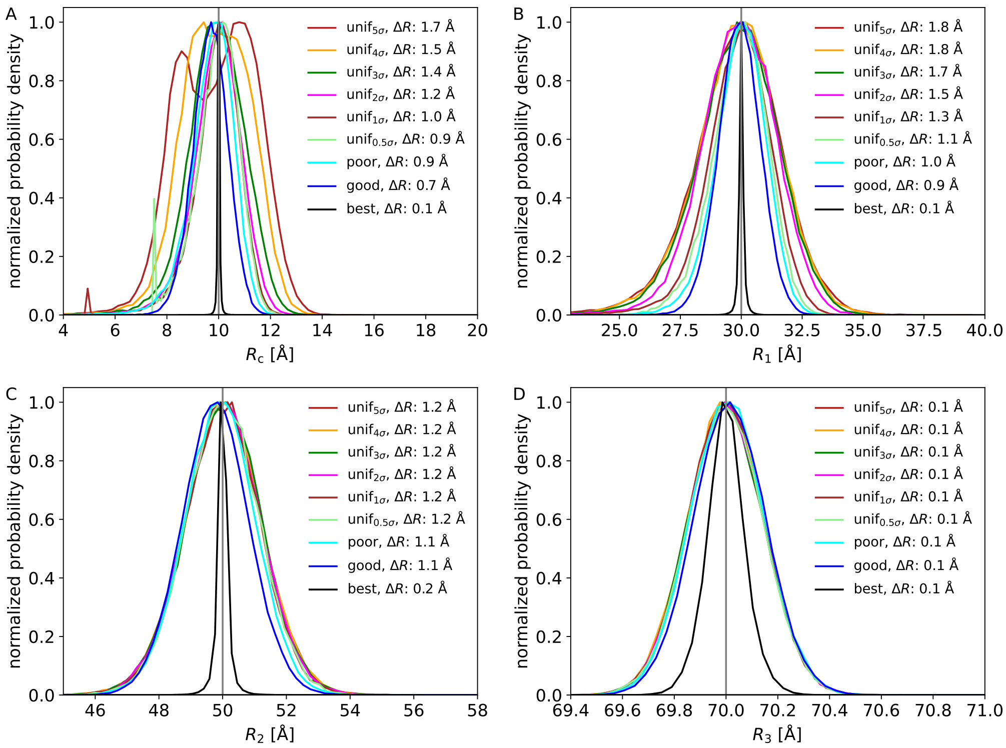

Fig. S8. Radii of the core-multishell model were refined against SAXS and SANS data, using a non-informative uniform prior (red), a poor Gaussian prior (light blue), a good Gaussian prior (dark blue) or the best Gaussian prior (black). The probability distributions were normalized with the maximum, for easier comparison. The model was also refined with more informative uniform priors with upper and lower limits set to $\mu_k \pm x\sigma_{\text{best}}$ and x is varied from 0.5 to 5, where $x = 5$ corresponds to the non-informative uniform prior. (A) Core radius, (B-D) outer radii of shells 1-3.
